# Enhancing Early Integration of Pediatric Palliative Care: Evaluating a Clinician-Based Versus Criteria-Based Referral Process in Pediatric Oncology

**DOI:** 10.3390/children13070927

**Published:** 2026-07-15

**Authors:** Alexis Fong-Leboeuf, Christina Vadeboncoeur, Donna L. Johnston

**Affiliations:** 1 Faculty of Medicine, University of Ottawa, Ottawa, ON K1H 8M5, Canada; 2 Roger Neilson Children’s Hospice, Ottawa, ON K1H 8L2, Canada; 3 Pediatric Palliative Care Program, Children’s Hospital of Eastern Ontario, Ottawa, ON K1H 8L1, Canada; 4 Division of Hematology/Oncology, Children’s Hospital of Eastern Ontario, Ottawa, ON K1H 8L1, Canada

**Keywords:** pediatric palliative care, early referral, pediatric oncology, quality improvement, referral criteria

## Abstract

**Highlights:**

**What are the main findings?**

- Formal criteria-based referral processes for new oncologic diagnoses support increased pediatric palliative care referrals.
- Formal criteria-based referral processes for all new oncologic diagnoses also resulted in earlier referrals, with decreased time between diagnosis and integration of pediatric palliative care.

**What is the implication of the main finding?**

- With appropriate infrastructure, oncology programmes may benefit from creating formal criteria-based referral processes for new oncologic diagnoses to encourage earlier integration and support of palliative care teams.

**Abstract:**

Background/Objectives: Early palliative care referral for new oncologic diagnoses has been widely recognized as integral to comprehensive care, but there exists a paucity of research assessing referral patterns in pediatric oncology. The objective of this study is to review the change in referral patterns at the Children’s Hospital of Eastern Ontario (CHEO) before and after a set of referral criteria were implemented for routine consultation of pediatric palliative care (PPC) for all newly diagnosed oncology patients. Methods: This was a retrospective single-centre chart review of all patients with a new oncologic diagnosis from 21 October 2017 to 1 January 2024 using information available in the patient’s electronic medical record. Data was evaluated for referral patterns and the time from diagnosis to referral to PPC comparing the period before and after referral criteria were initiated (1 July 2019). Results: A total of 504 chart reviews were completed and 226 patients were referred to PPC. The proportion of patients referred to PPC showed a statistically significant increase from 36.8% to 75.4% after introduction of PPC referral criteria (χ2(1) = 44.98, *p* < 0.001). The time from diagnosis to referral showed a statistically significant decrease from a median of 267 days (IQR 116–551.5) prior to criteria to 11 days (IQR 4–27) post-criteria (Mann–Whitney U test, *p* < 0.001). Time to referral decreased significantly across all diagnostic subgroups except for CNS tumours. Conclusions: Having referral criteria for consultation to PPC increased the proportion of oncology patients referred to PPC and resulted in a significant decrease in time to referral. These findings suggest that structured referral criteria support earlier integration of pediatric palliative care within oncology programmes.

## 1. Introduction

In 2020, there were over 200,000 new diagnoses of childhood cancer worldwide, with the incidence continuing to increase [1]. This growing patient population experiences heavy symptom burden, long treatment duration, and significant psychosocial challenges. Integral to the care of children with oncologic diagnoses, the utilization of pediatric palliative care (PPC) improves multiple aspects of care including, but not limited to, symptom burden, patient and family quality of life, and decreased hospitalizations [2]. The World Health Organization (WHO) defines PPC as *the prevention and relief of suffering of pediatric patients and their families facing the problems associated with life threatening illness, including physical, psychological, social, and spiritual suffering of patients and their family members* [3]. Ultimately, it is the goal of the PPC team to follow patients and their families throughout their illness trajectory, providing continuity of care on both an outpatient and inpatient basis. This is contrary to the historical misconception that palliative care is synonymous with end-of-life care, or hospice care.

Early palliative care referral for new oncologic diagnosis has been widely recognized as imperative to comprehensive care. Per the American Society of Clinical Oncology (ASCO), it is considered standard of care to refer all new advanced cancer diagnoses, and was most recently updated in 2024 to encourage referral as soon as possible [4]. While PPC is a relatively new subspecialty, it mimics many of the qualities of its adult counterpart. The Canadian Network of Palliative Care for Children recommends that “children and families have equal and timely access to pediatric hospice palliative care services” [5], values shared by the WHO [3] and American Academy of Pediatrics [6]. Lee et al. (2023) found that earlier PPC integration in the pediatric oncology population may be associated with improved code status documentation and that patients were less likely to receive intensive interventions (CPR/intubation) at the time of death [7]. Early integration in pediatric oncology patients enrolled in Phase 1 trials was associated with earlier hospice enrolment, decreased hospitalizations and intensive care utilization and better symptom management [8]. Despite this, a recent systematic review indicated that only 42.5% of pediatric oncology patients received any pediatric palliative care intervention prior to death [9].

There is little research in the PPC population outlining best practices to optimize outcomes for new oncologic diagnoses and timely referral. Historically, most palliative care programmes have operated within a “clinician-based” referral system, whereby referrals are initiated at the discretion of individual clinicians [10]—a practice that may be biassed by the personal characteristics, beliefs, and attitudes of the primary provider, which may influence timing of PPC consultation [11]. Some institutions refer via automatic referral criteria—for example, Golan et al. described routine referral of all children with cancer diagnoses with a less than 30% projected overall survival [12]. Initiating screening criteria in the pediatric critical care environment improved rates of referral to PPC [13], suggesting a similar approach may benefit the pediatric oncology population, though a recent expert consensus suggests that outpatient palliative care referrals should be based both off automatic (criteria-based) and clinician-based referrals [10]. At this time, there is minimal evidence to suggest or define the optimal referral practices to pediatric palliative care specifically in the pediatric oncology population, and none that explore outcomes after a change in referral practices in this population. A recent Canadian study explored different referral patterns of pediatric oncology patients to PPC through a Systems Theory Model; recommending the consideration of standardized referral criteria through interviewing of healthcare professionals, but does not quantitatively demonstrate its benefits [14].

Prior to 1 July 2019, all referrals to the PPC team at the Children’s Hospital of Eastern Ontario (CHEO) were initiated based on independent physician discretion, with the initiation of defined palliative care referral criteria for the oncology population thereafter. This study seeks to evaluate the efficacy of a mixed criteria-based and clinician-based referral practice for children with new oncologic diagnoses to the local PPC team, to assess if this resulted in increased referral rates and decreased time to referral from diagnosis.

## 2. Materials and Methods

CHEO serves over 500,000 children and youth from Eastern Ontario, Western Quebec, Nunavut, and Northern Ontario [15]. CHEO’s PPC programme was initiated in 1999, growing in size and expanding into a hospice adjacent to the hospital in 2006 [16]. Historically, oncology providers at CHEO utilized a clinician-based referral practice, relying on independent clinician discretion to determine who would benefit from a PPC referral. In July 2019, this switched to a mixed clinician-based and criteria-based referral practice, with referral criteria listed in Table 1, meaning that clinicians were meant to refer any new oncologic diagnosis that met referral criteria, in addition to anyone they felt might benefit at or shortly after diagnosis. These criteria were created through collaboration of physicians in the oncology and palliative care teams. This mixed model was utilized to capture any oncology patients who may not meet criteria, but were deemed by the physician to benefit from the service in order to help achieve the best quality of care. In addition, this would offer additional data that could be reviewed to inform what patient populations were referred via clinician-based referral and to feed back into future possible iterations of the automatic referral criteria.

Prior to practice change in July 2019, all oncologists and palliative care physicians and oncology nurse case managers were invited to a formal meeting to review this new formal referral process. There was no formal quality review of this process until this paper. Composition of both oncology and palliative care teams varied over the course of the study period.

Prior to data collection, permission from local REB was confirmed, establishing this project as a quality improvement initiative. A retrospective chart review was performed on all children from CHEO that received a new oncologic diagnosis, followed by the local pediatric hematology/oncology team from 21 October 2017 to 1 January 2024, with outcome data assessed until 8 April 2024. The date of 21 October 2017 was chosen as this was when electronic medical records were initiated fully at CHEO. Criteria-based referrals were initiated on 1 July 2019. Some of this patient information was already available through the local Pediatric Oncology Database—all patients in this database were included in initial data collection. Data not available in the database had to be collected from patient charts. Data was collected via a RedCap collection tool (reviewed by authors A.F.-L., C.V., D.L.J.) and de-identified prior to analysis. All data was collected by author A.F.-L. Clear guidelines and rules were established in the RedCap collection tool (available upon request) on how missing data was to be handled or extrapolated from other parts of the EMR, and ultimately there were no patients for which required data could not be found in the chart. Patients who moved/transferred during the study period were excluded as well as patients who were lost to follow-up.

### Analysis

**Figure 1:**
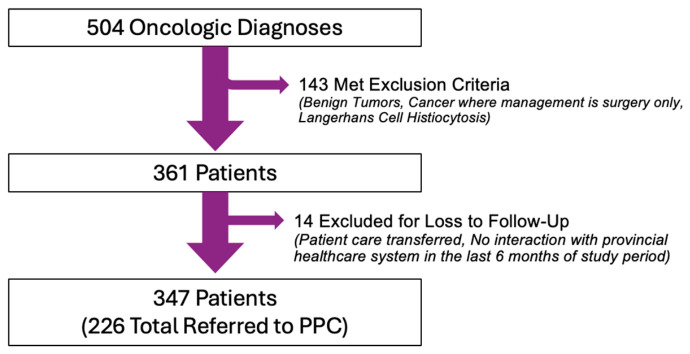
Participant flow diagram for inclusion or exclusion from study.

To be included in this study, a patient needed to receive a new oncologic diagnosis during the study period. All oncologic diagnoses referred to the hematology/oncology team were collected in a database. In theory, patients with benign tumours who were not referred to hematology/oncology may have been missed in the initial sampling, but would have been ultimately excluded. Patients were excluded per referral criteria in Table 1, if they were diagnosed with a benign tumour (any tumour not felt to be life-limiting, where management is observation-only), a cancer where management was surgery-only, or Langerhans cell histiocytosis. Other patients were later excluded if their outcomes could not be followed, or if they had diagnoses that did not explicitly meet referral criteria. Initial data cleaning and simple calculations were completed in Microsoft Excel with statistical analysis completed in R. Five hundred and four new oncologic diagnoses were made over the time period, with 143 meeting exclusion criteria per Figure 1, leaving 361 patients for data analysis. Fourteen patients were lost to follow-up, either having transitioned immediately to adult care or moved away during treatment. Of the remaining 347 patients, 226 were referred to PPC.

To assess overall general trends in referral to PPC of eligible oncologic patients before and after initiation of referral criteria, a run chart was utilized to visualize overall trends from a quality improvement standpoint, and chi squared test to assess for statistical significance. Data was analyzed utilizing R (Version 2026.01.0+392).

Time to referral (TTR) was then assessed, defined as the number of days from the date of oncologic diagnosis (defined by date of positive pathology) to the date the consult was placed to PPC, and compared in the pre- and post-criteria time frames using Mann–Whitney U test for two independent samples. This was also evaluated at a subgroup level evaluating TTR of patients subdivided into 3 categories: leukemia/lymphoma, CNS tumours, and solid tumours, in addition to looking specifically at patients who died. Patients with a hematologic cancer were allocated to the leukemia/lymphoma group, any intracranial malignancy was allocated to the CNS group, and all extra-cranial solid tumours were allocated to the solid tumour group based on the primary site of disease. See Table S1 for a breakdown of the diagnoses that fit into each of these categories after exclusions were removed.

## 3. Results

### 3.1. Demographics

There were 347 patients included in the study, having met protocol criteria for inclusion during the study period. Initial demographics of the full cohort of patients regardless of whether they had been referred to PPC is demonstrated in Table 2, and showed that age of patients at diagnosis ranged from 0 (birth) to 18.52 years. Average age at diagnosis was 8 years of age, though median age of diagnoses skewed slightly older in the pre-criteria cohort. Leukemia/lymphoma was the most common diagnosis, approximately half of all diagnoses, followed by solid tumours and then CNS tumours. During analysis a few diagnoses did not fit into other categories and were grouped as “Other”, including paraganglioma, squamous papilloma, malignant melanoma, and plexiform fibrohistiocytic tumour.

### 3.2. General Referral Patterns

**Figure 2:**
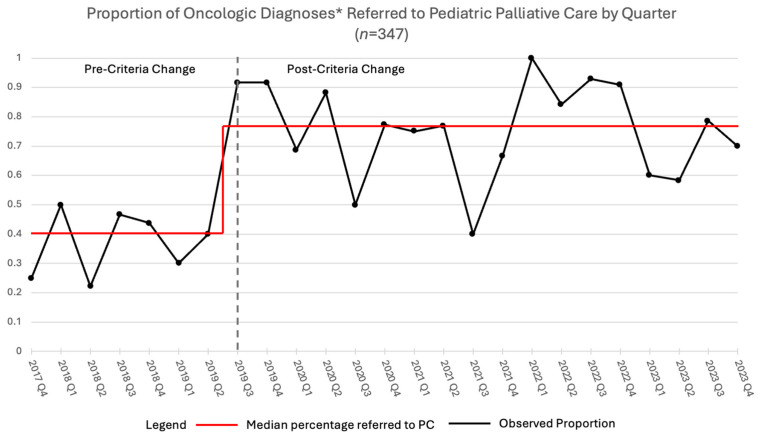
Proportion of oncologic diagnoses referred to pediatric palliative care by quarter (*n* = 347). * Calculated by comparing the number of patients referred to pediatric palliative care to the total number of patients who met the criteria to be referred to pediatric palliative care, visualized in the pre-criteria change and post-criteria change time periods. Dashed line indicates the point at which referral criteria were initiated.

Of the 347 patients included, 226 were referred to PPC. A run chart was utilized to visualize change in proportion of referrals to PPC before and after initiation of the referral criteria. In the pre-criteria period, the median proportion of referrals to PPC was 0.40, whilst in the post-criteria change period, the median proportion of referrals to PPC was 0.77. Utilizing quality improvement principles, a shift can be seen after the introduction of PPC referral criteria, visualized in Figure 2. The overall percentage of patients who met criteria for PPC referral and then received referral showed a statistically significant increase from 36.8% to 75.4% (χ^2^(1) = 46.09, *p* < 0.001, V = 0.36).

**Figure 3:**
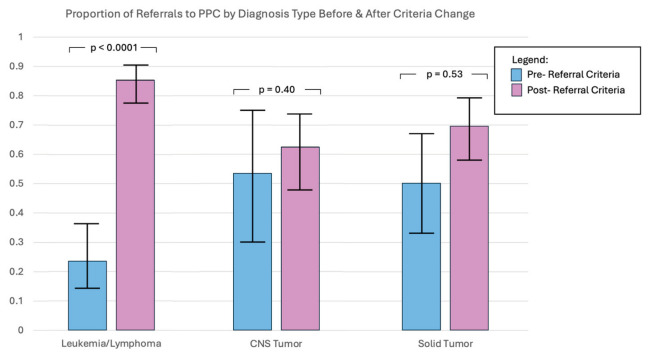
Proportion of patients meeting the referral criteria who were referred to PPC, divided into subtypes—leukemia/lymphoma, CNS tumours, and solid tumours—comparing the before and after referral criteria periods with 95% confidence intervals shown.

The patients were categorized into three distinct oncologic diagnosis categories—leukemia and lymphoma, CNS tumours, and solid tumours—to assess the differences in compliance with PPC referral (3.2) and trends in time to referral (3.3.2). There was a small subset of patients who did not fall into these three categories and were excluded for subset analysis. When analyzed by diagnoses, there was a statistically significant increase in proportion of leukemia/lymphoma referrals to PPC, increasing from 0.24 to 0.85 of patients who met criteria (χ^2^(1) = 67.40, *p* < 0.001, V = 0.61). While there was an increase, it was not a statistically significant increase in the proportion of patients referred with CNS tumours (χ^2^(1) = 0.40, *p* = 0.53, V = 0.08) or solid tumours (χ^2^(1) = 3.31, *p* = 0.06, V = 0.18), as shown in Figure 3.

#### 3.2.1. Time from Oncologic Diagnosis to PPC Referral

**Figure 4:**
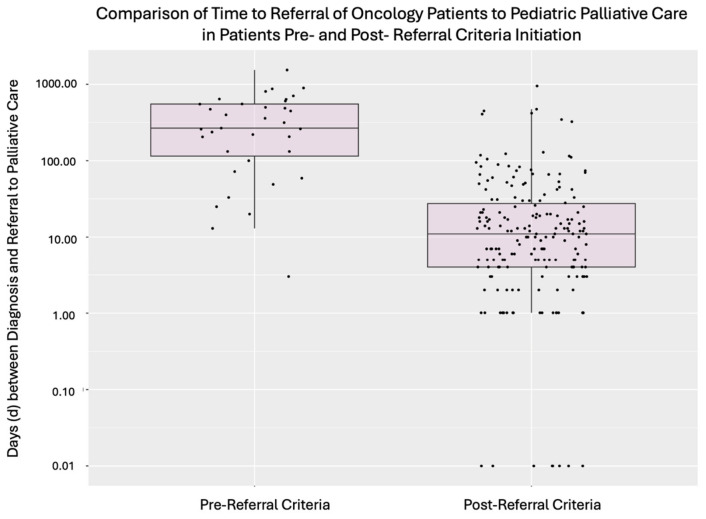
Comparison of time to referral (TTR) in days of oncology patients referred to pediatric palliative care, comparing patients pre- and post- referral criteria initiation.

Looking specifically at only those who were referred to PPC (*n* = 226), time to referral was calculated (in number of days (d) between date of diagnosis and date of referral to PPC) in both the pre- and post-criteria cohort. Thirty-five patients were referred to PPC in the pre-criteria cohort and 191 patients in the post-criteria cohort. Figure 4 demonstrates the statistically significant decrease in time to referral of oncology patients to PPC, from a median of 267 days to 11 days (Mann–Whitney U test, *p* < 0.001, r = 0.52). Twenty-eight (14.5%) of the patients in the post-criteria group were still referred over 8 weeks after oncologic diagnosis, with one patient being referred to PPC 950 days after diagnosis.

#### 3.2.2. Time from Oncologic Diagnosis to PPC Referral—Diagnosis Subgroups

**Figure 5:**
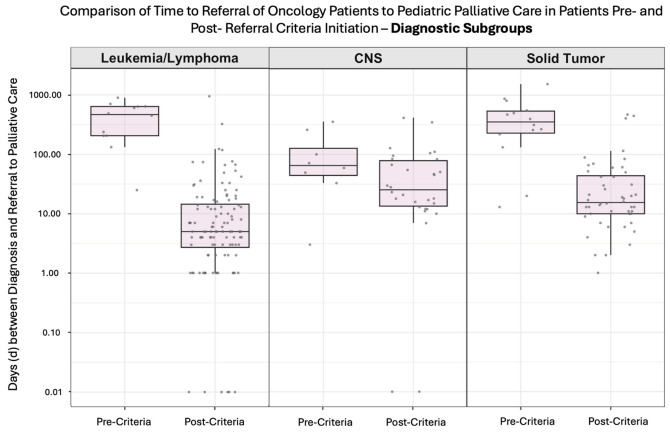
Comparison of time to referral (TTR), in days, for oncology patients referred to pediatric palliative care, comparing diagnostic subgroups of leukemia/lymphoma (pre = 12, post = 113), CNS tumours (pre = 8, post = 30), and solid tumours (pre = 14, post = 48).

Once categorized into three distinct diagnostic categories (leukemia/lymphoma, CNS tumours, and solid tumours), time to referral (in days) was compared during the pre- and post-referral criteria period to assess a decrease in TTR, as demonstrated in Table 3, and Figure 5. Leukemia/lymphoma patients (Mann–Whitney U test, *p* < 0.001, r = 0.48) and solid tumour patients (Mann–Whitney U test, *p* < 0.001, r = 0.57) were found to have significantly decreased TTR after the initiation of referral criteria, though CNS tumours showed no significant decrease in TTR (*p* = 0.13).

#### 3.2.3. Time from Oncologic Diagnosis to PPC Referral–Deceased Patients

**Figure 6:**
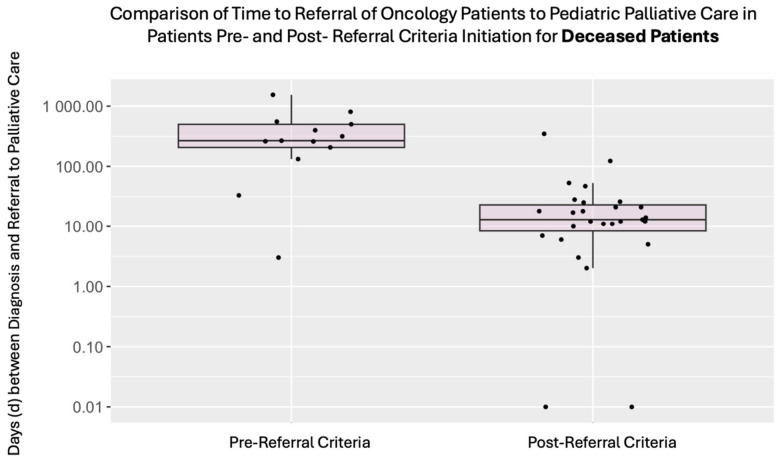
Comparison of time to referral (TTR), in days, for oncology patients referred to pediatric palliative care, comparing patients pre- (*n* = 13) and post-referral criteria initiation (*n* = 27), examining the subgroup of patients who died during the study period.

TTR was evaluated in the specific subgroup of patients that died during the study period (this included any patient that met the study inclusion criteria and who were then referred to PPC) and shown in Figure 6. There was a significant decrease in TTR after the initiation of referral criteria from a median of 267 to 13 days (*p* < 0.01, r = 0.65).

## 4. Discussion

This study aimed to evaluate the impact of referral criteria for pediatric palliative care in the pediatric oncology population. As expected, the introduction of specific referral criteria significantly increased the proportion of patients that were referred to pediatric palliative care in addition to significantly decreasing time to referral.

### 4.1. Increased Referral Rates

Criteria-based referrals may decrease barriers to consultation such as: family “readiness”, family preferences, and diagnostic uncertainty [17]. The utilization of referral criteria should reinforce referral to PPC as a “standard-of-routine-care”, minimizing these factors. Physician-specific barriers surrounding palliative care referral include discomfort discussing death/palliative care, as palliative care is often conflated with end-of-life care [18,19]. Integration of routine referral may help destigmatize these conversations; however the barriers preventing higher referral rates remain unclear and warrant further investigation. Some of this fluctuation may be impacted by the number and type of oncologic diagnoses made each month, as there may be periods during which more solid tumour or CNS tumours are diagnosed (which showed an insignificant change in the proportion referred) or fewer oncology diagnoses overall.

When subcategorizing patients into diagnostic groups, there was an increase in proportion of leukemia/lymphoma patients referred, but there was not a significant change in the CNS tumour and solid tumour group. The absence of a statistically significant increase in referral proportions for CNS and solid tumour groups likely reflects high baseline referral rates in these groups prior to criteria implementation, limiting measurable improvement. Previously patients with low-risk leukemia were not referred to PPC at all (allowing for more opportunity for improvement), whereas CNS and solid tumour patients were more likely to be referred to PPC based on clinician choice (thus potentially leaving less room for improvement). While not explored in the context of this study, a 2021 US study showed that oncologists most often referred patients to PPC for specific disease types, poor prognosis, high risk or relapsed disease, intense therapy, or significant symptoms [18]. The increase in referrals for leukemia/lymphoma patients (a subgroup of patients felt to have generally favourable prognosis) will be important to evaluate through cost–benefit analysis to determine whether this is reproducible or feasible in smaller programmes and to assess the specific benefits to patients and families. At present, there is no evidence of routine PPC consultation for all leukemia/lymphoma patients in other Canadian institutions. While this study shows the process outcome of increased and timely referrals, further research should explore family and patient outcome measures to help determine where automatic referral is most beneficial.

### 4.2. Time to Referral

Median time to referral after diagnosis also significantly decreased after initiation of the referral criteria, from 267 days to 11 days, though there was still moderate variability, with 14.5% of patients in the post-criteria population having not been referred to PPC by 8 weeks after oncologic diagnosis despite the previous recommendation from the ASCO. It is unclear what barriers contributed to late or missed referrals overall, with room for further research at this specific institution.

Earlier integration of pediatric palliative care may have a significant impact on patient and family quality of life. Earlier referral to palliative care teams allows for more cumulative exposure and opportunities for relationship building and symptom management during treatment. Patients who survive cancer are at high risk of both cognitive difficulties and increased psychosocial burdens, but protective factors, including processes/resources that buffer stress, support adaptive functioning, and promote resilience across the life span, may help with the sequelae of therapy [20].

When evaluating TTR of the patients by diagnostic group, it was noted that there was a decreased TTR for both solid tumour and leukemia/lymphoma patients but not CNS tumours. Previously, referrals may have been initiated in the context of sentinel events, such as symptom management or disease recurrence, occurring later in the disease course. With the change to referral soon after diagnosis, a decrease in TTR is expected. The CNS tumour subgroup of patients did not experience a significant decrease in TTR after diagnosis. This may be in part to the rapid referral of these patients to surgery for biopsy and subsequent radiotherapy, which does not always occur at the study hospital, with patients sometimes travelling to the United States for proton radiation therapy early in the disease course. Additionally, differences in TTR between oncologic subtypes were noted. CNS tumours had the shortest TTR in the pre-criteria period, which left less room for improvement. This again was likely related to disease prognosis, symptom burden, and clinician practices, but should be interpreted carefully as some subgroup analyses were underpowered.

An additional subgroup of patients that were investigated were the patients who died during the study period. Unfortunately, this subgroup was also limited by its small numbers in the pre-criteria period. With the change in referral practice, there was a substantial improvement in TTR for this patient population from 267 days to 13 days after diagnosis. The TTR of the patients that died (13 days) was slightly higher than the median TTR of the overall group (11 days), which included patients with very good prognoses. This further supports that ongoing research is needed to evaluate barriers to timely referral.

### 4.3. Limitations/Confounding Variables

The retrospective nature of this study introduces several potential confounding factors, including the possibility that general awareness of PPC may have increased over the study period and variables such as variation in oncology and palliative care team composition. Limitations to subgroup analysis include the low number of participants in the pre-criteria period, impacting the ability to detect statistical significance. The decision was made to start data analysis from the date the electronic medical record was available for standardization of data collection; however collecting pre-EMR data may have increased statistical power. Furthermore, the documentation of data in the EMR may be inconsistent across providers. In addition, when subcategorized into diagnoses, some analyses were underpowered, limiting interpretation.

### 4.4. Strengths

While the retrospective nature of this study is a limitation, it also offers real-world clinical evidence that a mixed criteria and physician-based referral process increased referrals in addition to more timely referrals. Single-centre design also allowed for the evaluation of practice change within a single setting, reducing variability in team composition and hospital care models. The inclusion of both referral rates and time to referral also provides a more holistic understanding of potential impacts on the referral process.

### 4.5. Clinical Implications and Future Directions

While the goal of this practice change was to encourage early integration of pediatric palliative care into oncology practice, the question arises regarding the functional impact on patients and their families, in addition to the cost analysis to the system. While early integration is considered standard of care, it is not clear at this time which pediatric oncology families benefit the most from this early integration and whether this is feasible for all palliative care programmes. Automatic referral of oncology patients to palliative care has been described as “more of a vision than a reality”, as many palliative care teams are not equipped to handle such large patient populations [21]. For this reason, individual institutions may need to strike the independent balance of inclusive criteria, whilst limiting expansion beyond local capacity. To our knowledge, the Children’s Hospital of Eastern Ontario PPC program is the only program in Canada that is referred most oncologic diagnoses; including all pediatric leukemia patients, who typically have favourable prognoses. Further research is required to assess the impacts of PPC integration in this specific subset of oncology patients, in addition to its reproducibility to other programmes.

## 5. Conclusions

Early palliative care referral for patients with a new oncologic diagnosis has been widely recognized as integral to comprehensive care. Utilization of referral criteria (including physician discretion) helps support increased and earlier referrals overall. Further research is needed to better understand the functional impacts of earlier and more broad referrals on families, in addition cost–benefit analysis, resource utilization, and subsequent feasibility for different programmes.

## Figures and Tables

**Table 1:** July 2019 criteria for automatic referral of patients with new oncologic diagnoses to PPC at CHEO.

Criteria for Automatic Referral to the Pediatric Palliative Care Team
Standard-risk leukemia (age 1–10 years, WBC < 50,000 mm^3^ at diagnosis, no CNS or testicular disease, no unfavourable cytogenetic features). Wilm’s tumour. Hodgkin’s lymphoma. Brain tumours. High-risk leukemia. Solid tumours. Any child whom hematology–oncology or pediatric palliative care feels might benefit from referral.
Excluded:
Benign tumours. Cancer where management is surgery only. Langerhans cell histiocytosis.

**Table 2:** Demographics of all patients that met appropriate criteria for analysis, having been referred to oncology for a new oncologic diagnosis during the protocol period.

Demographics	Overall (*n* = 347)	Pre-Criteria (*n* = 95)	Post-Criteria (*n* = 252)	*p* Value
**Age at diagnosis, median (IQR), years**	7.07 (3.41–13.78)	8.02 (3.70–13.78)	7.05 (3.36–14.04)	0.942
**Gender**				
**Male (proportion)**	190 (0.55)	50 (0.53)	140 (0.56)	0.626
**Female (proportion)**	157 (0.45)	45 (0.47)	112 (0.44)	0.626
**Diagnoses (proportion)**				
**CNS Tumors**	63 (0.18)	15 (0.16)	48 (0.19)	0.469
**Leukemia/Lymphoma**	182 (0.52)	51 (0.54)	131 (0.52)	0.813
**Solid Tumor**	97 (0.28)	28 (0.29)	69 (0.27)	0.719
**Other**	5 (0.01)	1 (0.01)	4 (0.02)	n/a

**Table 3:** Subgroup analysis of oncology patients, examining median time to referral (in days) in diagnostic subgroups (leukemia/lymphoma, CNS tumours, solid tumours) and in deceased patients, and associated *p*-value for statistical significance using Mann–Whitney U test for independent samples.

Group Analysis	Pre-Criteria	Post-Criteria	*p* Value
**Overall (*n*)**	35	191	
**Mean TTR, days**	373.3	37.5	
**Median TTR, days (IQR)**	267.0 (116.0–551.5)	11 (4–27)	<0.001
**Diagnosis Subgroups:**			
**CNS Tumors (*n*)**	8	30	
**Mean TTR, days**	117.0	62.9	
**Median TTR, days (IQR)**	65.5 (45.0–140.3)	25.5 (13.5–79.0)	0.13
**Leukemia/Lymphoma (*n*)**	12	113	
**Mean TTR, days**	434.7	14.0	
**Median TTR, days (IQR)**	466.5 (205.8–636.5)	5 (3.0–14.0)	<0.001
**Solid Tumors (*n*)**	14	48	
**Mean TTR, days**	454.4	51.8	
**Median TTR, days (IQR)**	356.5 (229.8–539.5)	15.5 (10.0–44.0)	<0.001
**Deceased Patients (*n*)**	13	27	
**Mean TTR, days**	405.3	31.9	
**Median TTR, days (IQR)**	267 (206.0–499.0)	13 (8.5–23.0)	<0.01

## Data Availability

The data presented in this study are available on request from the corresponding author, as, while every effort has been made to de-identify the data, given the small local patient population, sharing the full data set may still be identifying.

## Supplementary Materials

The following supporting information can be downloaded at: https://www.mdpi.com/article/10.3390/children13070927/s1, Table S1: Included diagnoses and which subgroups they fell under for analysis, when grouped into CNS tumors, Leukemia/Lymphomas, Solid Tumors, or Other.

## Abbreviations

The following abbreviations are used in this manuscript:

PPC	pediatric palliative care
TTR	time to referral
ASCO	American Society of Clinical Oncology
