# Supplementary material for: Enhancing Early Integration of Pediatric Palliative Care: Evaluating a Clinician-Based Versus Criteria-Based Referral Process in Pediatric Oncology
Source: Children (Basel). 2026 Jul 15;13(7):927. doi: 10.3390/children13070927 (PMC13406938; doi:10.3390/children13070927)
Supplement: Supplementary file 1 [file children-13-00927-s001.zip › children-4379024-supplementary.pdf]

CNS Tumors	Leukemia/Lymphoma	Solid Tumor	Other
Anaplastic High Grade Ependymoma	Acute Megakaryoblastic Leukemia	Adenocarcinoma	Malignant Melanoma
Astroblastoma	Acute Myeloid Leukemia	Adrenocortical Carcinoma	Monomorphic Lymphoproliferative Disease (PTLD) - DLBCL
Astrocytoma (Pilocytic, Protoplasmic, etc.)	Acute Myelomonocytic Leukemia	Atypical Teratoid Rhabdoid Tumor (Renal)	Paraganglioma (Malignant) - Extra Adrenal Pheochromocytoma
Brain Sarcoma	Acute Promyelocytic Leukemia	Clear Cell Carcinoma (Renal)	Plexiform Fibrohistiocytic Tumor
Brainstem Glioma	Burkitt's Lymphoma	Desmoplastic Small Round Cell Tumor	Squamous Papillomatosis
Diffuse Intrinsic Pontine Glioma	Chronic Myeloid Leukemia	Dysgerminoma (Ovarian)	
Ependymoma	Hodgkin Lymphoma	Embryonal Carcinoma (Testicular)	
Ewing's Sarcoma (CNS)	Hodgkin Nodular Sclerosis	Embryonal Sarcoma	
Germinoma Pineal	Large Cell Peripheral T-Cell Lymphoma	Endodermal Sinus / Yolk Sac Tumor (Non-Gonadal)	
Glioblastoma Multiforme	Lymphoblastic Lymphoma (Precursor B-Cell)	Endodermal Sinus / Yolk Sac Tumor (Ovarian)	
Malignant Rhabdoid Tumor (CNS)	Lymphoblastic Lymphoma (Precursor T-Cell)	Epithelioid Sarcoma	
Medulloblastoma (Desmoplastic Nodular, etc)	Mediastinal Large B-Cell Lymphoma	Ewing's Sarcoma	
Melanomatosis (Meningeal)	Mixed Phenotype Acute Leukemia (B/Myeloid)	Fibromatosis / Desmoid Tumor	
Mixed Germ Cell Tumor (CNS)	Mixed Phenotype Acute Leukemia (T/Myeloid)	Hepatoblastoma	
Optic Pathway Glioma	NHL Large Cell Lymphoma Anaplastic T Cell / Null Cell	Infantile Fibrosarcoma	
Retinoblastoma	Polymorphic Lymphoproliferative Disease (PTLD)	Juvenile Granulosa Cell Tumor	
Rhabdoid Tumor (CNS)	Precursor B-Cell Lymphoblastic Leukemia	Mixed Germ Cell Tumor (Ovarian)	
Teratoma (Mature, Immature, etc.)	Precursor B-Cell Lymphoblastic Lymphoma	Mixed Germ Cell Tumor (Testicular)	
Tectal Glioma	Precursor T-Cell Lymphoblastic Leukemia	Nasopharyngeal Carcinoma	
	Precursor T-Cell Lymphoblastic Lymphoma	Neuroblastoma	
		Osteosarcoma	
		PNET - Ewing's Sarcoma	
		Rhabdoid Tumor (Non-Kidney)	
		Rhabdomyosarcoma (Spindle Cell)	
		Rhabdomyosarcoma Alveolar	
		Rhabdomyosarcoma Embryonal	
		Synovial Sarcoma	
		Undifferentiated Sarcoma	
		Wilm's Tumor	

Table S1. Included diagnoses and which subgroups they fell under for analysis, when grouped into CNS tumors, Leukemia/Lymphomas, Solid Tumors, or Other.
